# Supplementary material for: Tread lightly interpreting group differences in genetic risk
Source: ArXiv. 2026 May 22:arXiv:2605.23164v1. Preprint. [Version 1] (PMC13228795)
Supplement: Supplement 1 [file NIHPP2605.23164v1-supplement-1.pdf]

Supplement

Methods

Simulations for expected genetic values: Using SLiM [25], we simulate a polygenic trait forward in time under neutral evolution, stabilizing selection, and divergent selection. First, we initialized a (burn-in) randomly mating population of 10,000 diploids for 40,000 generations with 10 chromosomes, each of 100kb, mutation rate of 1×10^{-07}, and recombination rate of 1×10^{-08}. Next, we split this 'ancestral' population into two populations of equal size (10,000) and let them evolve for the next 1,000 generations. The effect of new mutations on the trait were drawn from $\mathcal{N}(\mu_\beta = 0, \sigma^2_\beta = 0.005)$, following [60]. We model selection on the individual level with Gaussian fitness functions. Briefly, the relative fitness of an individual with trait value x in an environment with an optimum value (fitness peak) of μ_o and variance of ω_o is $W(x|\mu_o, \omega_o) = exp(-\frac{x-\mu_o}{2\omega_o})$. For a trait under stabilizing selection, the fitness function becomes $\mathcal{N}(\mu_o = 0, \omega_o = 16)$ before and after the population split. To simulate a trait under divergent selection the fitness function is the same as stabilizing selection during the 40,000 generation burn-in, and changes to $\mathcal{N}(\mu_o = -2.0, \omega_o = 16)$ and $\mathcal{N}(\mu_o = 2.0, \omega_o = 16)$ for populations 1 and 2, respectively, after the split. For a neutral trait, the fitness is uniform, i.e., independent of trait value. Each evolutionary scenario was replicated 100 times. To calculate the proportion of genetic variance due to allele frequency differences, we computed $\frac{\sum_{i=1}^{m} \beta_i^2 (f_i^1 - f_i^2)^2}{V_g}$; where V_g is the total genetic variance in the two-population system, m is the total number of causal loci, β_i is the effect size of the ith locus, f_i^1 and f_i^2 are the allele frequencies in populations 1 and 2, respectively [30]. V_g was calculated as the variance of the genetic vlaues, g. To compute the proportion of genetic variance due to LD, we first calculated the genetic variance between populations as $V_{gb} = \frac{(\bar{g_1} - \bar{g_2})^2}{4}$ and then subtracted out the variance due to allele frequency: $\frac{V_{gb} - \sum_{i=1}^{m} \beta_i^2 (f_i^1 - f_i^2)^2}{V_g}$; where $\bar{g_1}$ and $\bar{g_2}$ are the mean genetic values for populations 1 and 2, respectively.

Correlations between genetic ancestry and binary traits: To illustrate the pattern of correlation between health-related traits and genetic ancestry among admixed Americans, we downloaded Table S4 from [72]. This table reports the association between proportion of African ancestry and 1,191 traits among 8,311 individuals with mixed African and European ancestry from the Penn Medicine Biobank. To fit this association, the authors used logistic regression for binary traits (phecodes) and linear regression for quantitative traits with nuclear ancestry, mtDNA ancestry, the interaction between the two, and sex, age, and age^2 as the independent variables. From this set, we retained the results of 657 binary traits which had at least 100 cases.

PGS calculation in 1000 Genomes Project: We obtained high coverage 1000 Genomes Project (TGP) in VCF format [11] from `https://ftp.1000genomes.ebi.ac.uk/vol1/ftp/data_collections/1000G_2504_high_coverage/working/20220422_3202_phased_SNV_INDEL_SV/`, and converted the data to PLINK2 format (`www.cog-genomics.org/plink/2.0/`) [14]. We restricted the samples to 2,504 unrelated individuals commonly present in earlier releases of TGP. A skin pigmentation score weight file (PGS002110 [54]) was downloaded from PGS Catalog (`https://www.pgscatalog.org`) [36], and applied to all individuals for score calculation through ESCALATOR (`https://github.com/menglin44/`

ESCALATOR). 275,451 out of 275,831 variants from the original model ended up in score calculation, after the harmonization step excluding 6 variants with mismatched allele codes and 374 variants missing in TGP.

textbfAncestry calibration of PGS: Principal component analysis was performed using PLINK2 –pca command [14, 55], after pruning out variants with missing rate >5%, minor allele frequency <1%, LD >0.1 (–indep-pairwise 500 125 0.1), and aggregating markers across autosomes. We calibrated the original PGS values by modeling PGS mean and variance as a linear function of the top 5 PCs following methods by [31, 23] (see code availability). Specifically, PRS values can be modeled as $PGS_{obs} = \alpha_0 + \sum \alpha_i PC_i + \epsilon$, where the residual ϵ is equivalent to mean-calibrated PGS; further, variance is modeled as $\epsilon^2 = \beta_0 + \sum \beta_i PC_i + \epsilon'$, with mean and variance calibrated score as $\frac{PGS_{obs}-(\alpha_0+\sum \alpha_i PC_i)}{\sqrt{\beta_0+\sum \beta_i PC_i}}$.

Supplemental Figures

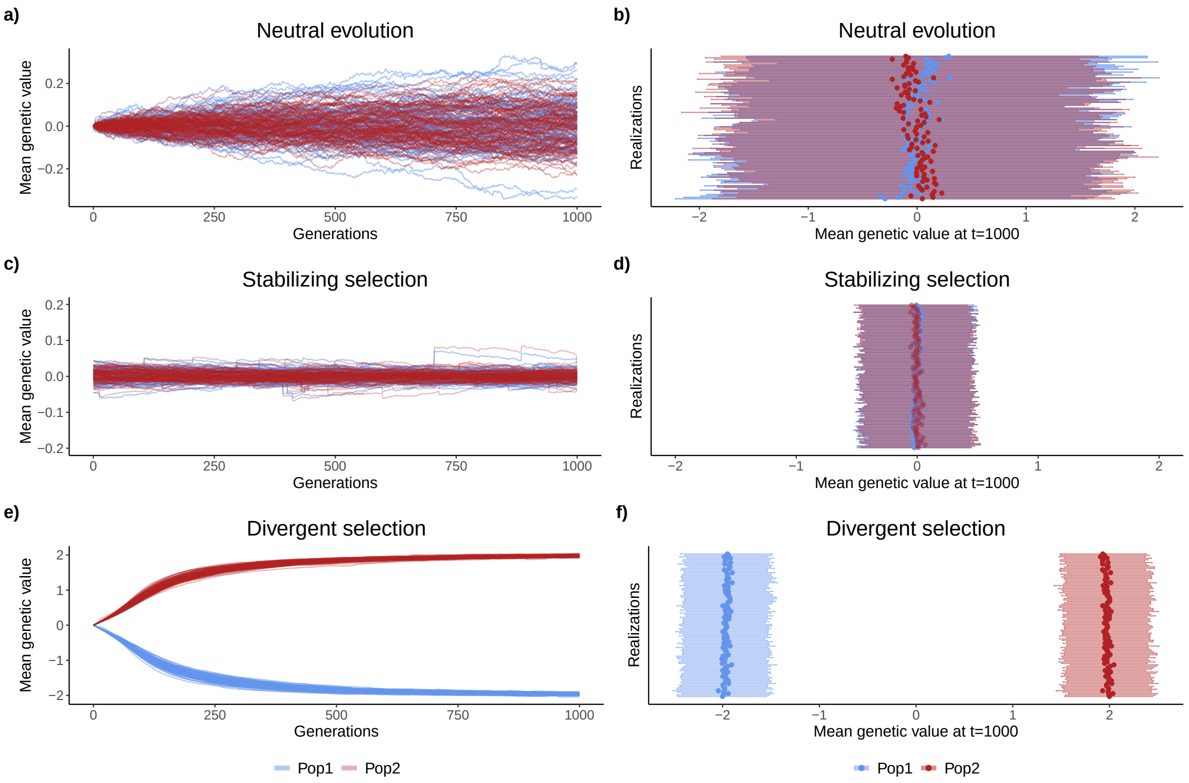

Figure S1: **Mean genetic values of two populations across 1,000 generations for traits under different evolutionary scenarios.** (**a, c, e**) An ancestral population of 10,000 individuals splits into two populations of equal size (10,000) after 40,000 generations, then the selection type continues to act of the polygenic trait for an additional 1,000 generations. (**b, d, f**) At generation 1,000, the mean genetic value for two populations (population 1 in blue and populations 2 in red) ordered by the difference in magnitude between the two populations genetic values across 100 realizations with 95% confidence intervals.
